# Type 1 Autoimmune Pancreatitis Masquerading as Pancreatic Head Carcinoma

**DOI:** 10.7759/cureus.47471

**Published:** 2023-10-22

**Authors:** Adesola A Agboola, Khalid H Mohamed, Maria Syed, Sheena Shiwlani, Rowaida Butt, Rezaur Rahman Reza, Muhammad Haseeb, Hira Nasir

**Affiliations:** 1 Pathology and Laboratory Medicine, Dele Hospitals, Lagos, NGA; 2 Neurology, Sheffield Teaching Hospitals NHS Foundation Trust, Sheffield, GBR; 3 Surgery, Aga Khan University Hospital, Karachi, PAK; 4 Pathology, Mount Sinai Hospital, New York, USA; 5 Family Medicine, Avalon University School of Medicine, Ohio, USA; 6 Internal Medicine, Jalalabad Ragib Rabeya Medical College, Lahore, PAK; 7 Internal Medicine, Allama Iqbal Medical College, Lahore, PAK; 8 Internal Medicine, Bahria International Hospital, Lahore, PAK; 9 Internal Medicine, Mayo Hospital, Lahore, PAK

**Keywords:** autoimmune pancreatitis, obstructive jaundice, pancreatic cancer, endoscopic ultrasound, immunosuppressive therapy

## Abstract

Obstructive jaundice is a joint clinical presentation with many etiologies, including pancreatic cancer and autoimmune pancreatitis (AIP). Differentiating between these two conditions is pivotal due to the divergent management approaches and prognoses. In this case report, we present a case of a 49-year-old female patient who presented with weight loss, intermittent chronic abdominal pain, and jaundice. She was initially suspected of having pancreatic cancer because of clinical presentation and imaging findings. However, she was ultimately diagnosed with Type 1 AIP due to histopathology findings and elevated immunoglobulin G4. This case highlights the complexities in diagnosis, the role of advanced imaging techniques and tissue sampling, and the lessons learned regarding managing this challenging clinical scenario.

## Introduction

Obstructive jaundice can result from various causes, including biliary obstruction, gallstones, tumors, and autoimmune diseases. Among the potential causes of obstructive jaundice, pancreatic cancer and autoimmune pancreatitis (AIP) pose diagnostic challenges due to their overlapping clinical and radiological features [1]. AIP is a rare form of chronic pancreatitis characterized by dense infiltration of lymphocytes and plasma cells, along with fibrosis triggered by an autoimmune response [2]. Given its rarity, the global prevalence of AIP stands at less than 1% per 100,000 individuals each year. AIP is mainly manifested in middle-aged to older patients, with a higher incidence in males as compared to females [3]. The clinical presentation of AIP is similar to that of pancreatic cancer, making it challenging to distinguish it from pancreatic carcinoma. It is crucial to distinguish between these two conditions promptly, as the management and prognosis are dramatically different [4]. We report a case of Type 1 AIP masquerading as pancreatic head carcinoma.

## Case presentation

A 49-year-old female with a past medical history of Hashimoto's hypothyroidism presented with intermittent abdominal pain for the last two months. Abdominal pain was generalized and worsened with eating. She also complained of progressive yellowishness of her skin and eyes, pruritis, and dark-colored urine associated with undocumented weight loss. She was diagnosed with Hashimoto's hypothyroidism ten years ago and was compliant with her medication, levothyroxine. She reported no history of constipation or diarrhea. She reported no history of alcohol, smoking, or any illicit drug use. She underwent an uncomplicated appendectomy when she was 14.

**Figure 1:**
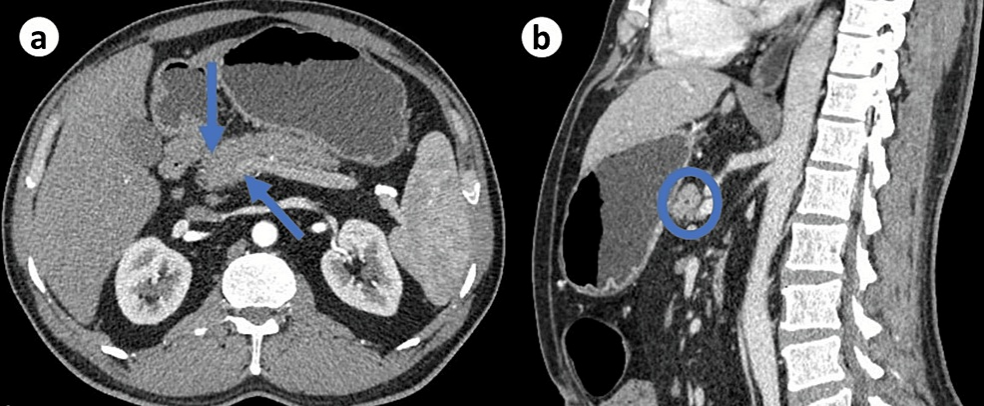
CT abdomen showing pancreatic head enlargement with irregular borders (a) and concentric thickening of the pancreatic duct (b). CT: computed tomography.

She was hemodynamically stable, afebrile, and visibly jaundiced with icteric sclera and generalized scratch marks. On abdominal examination, mild tenderness was noted in the epigastric region without any palpable mass or organomegaly. The rest of the systemic examination was unremarkable. Initial laboratory investigations revealed hemoglobin of 11.9 g/dl (reference range: 11.5-16), total bilirubin of 4.2 mg/dl (reference range: 0.2-1.2), direct bilirubin of 2.8 mg/dl (reference range: <0.2), alkaline phosphatase of 320 IU/L (reference range: 40-150), alanine aminotransferase of 45 IU/L (reference range: 7-56), aspartate aminotransferase of 38 IU/L (reference range: <40). Renal function tests were within normal limits. Serum lipase and amylase were within normal range with normal serum albumin levels. She underwent a contrast-enhanced computed tomography (CT) scan of the abdomen, which revealed a focal pancreatic head mass measuring around 4cm in diameter with irregular pancreas border and pancreatic and extrahepatic biliary duct dilation with concentric thickening (Figure 1). No lymphadenopathy was detected. The initial clinical presentation of weight loss, jaundice, and epigastric pain, coupled with the radiological finding of a pancreatic head mass, raised concerns about the possibility of pancreatic cancer. Given these alarming clinical and imaging features, the patient was scheduled for further evaluation.

**Figure 2:**
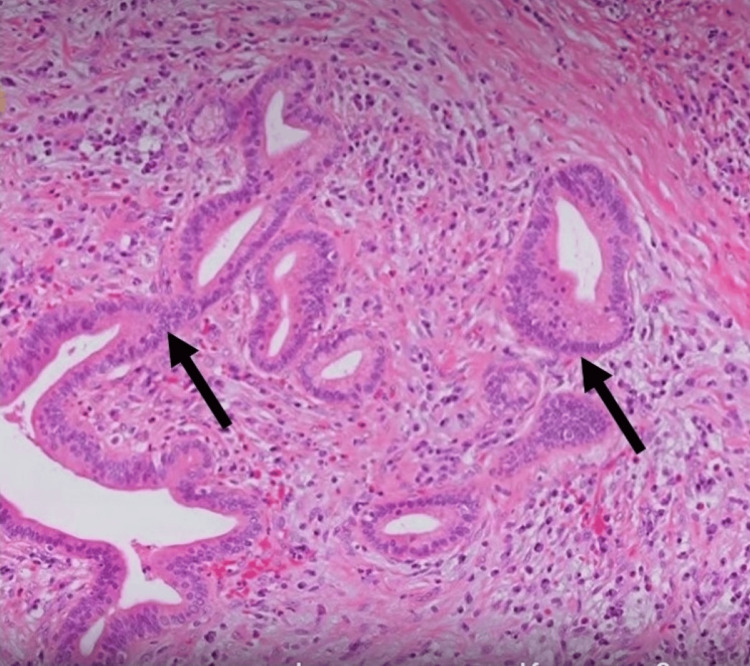
Biopsy of the pancreatic head lesion manifesting lymphocytic infiltrates with obliterative phlebitis suggestive of autoimmune pancreatitis.

She underwent an endoscopic ultrasound of the abdomen, which revealed bulky pancreatic parenchyma and hypoechoic with segmental stenosis of the pancreatic duct in the pancreatic head and extrahepatic duct narrowing with bile duct stricture. Peripancreatic fluid was not noted. She underwent fine-needle biopsy (FNB) with a 22G needle, which revealed a hypoechoic pancreatic head mass with an irregular border, causing biliary obstruction. FNB of the mass was carried out, and cytological analysis demonstrated abundant lymphoplasmacytic infiltration and fibrosis without parenchymal tissue involvement, which raised suspicion of autoimmune pancreatitis (Figure 2). In order to relieve obstructive jaundice, an endoscopic retrograde cholangiopancreatography was performed, and a biliary stent 0f 7.0 Fr was placed at the level of biliary stricture. Serological markers, including immunoglobulin (Ig) G4 levels, were measured, which were markedly elevated at 780 mg/dL (reference range: <135 mg/dL), and carbohydrate antigen (CA) 19-9 level was 44 IU/ml (reference range: <37 IU/ml) providing further evidence in favor of Type 1 AIP. Further autoimmune screening was negative, including anti-smooth muscle antibodies (SMA) and antinuclear antibodies (ANA).

She was promptly initiated on prednisone 40 mg daily, and there was a noticeable improvement in her symptoms within a week, including resolution of jaundice, reduction in abdominal pain, and cessation of pruritus.

## Discussion

**Figure 3:**
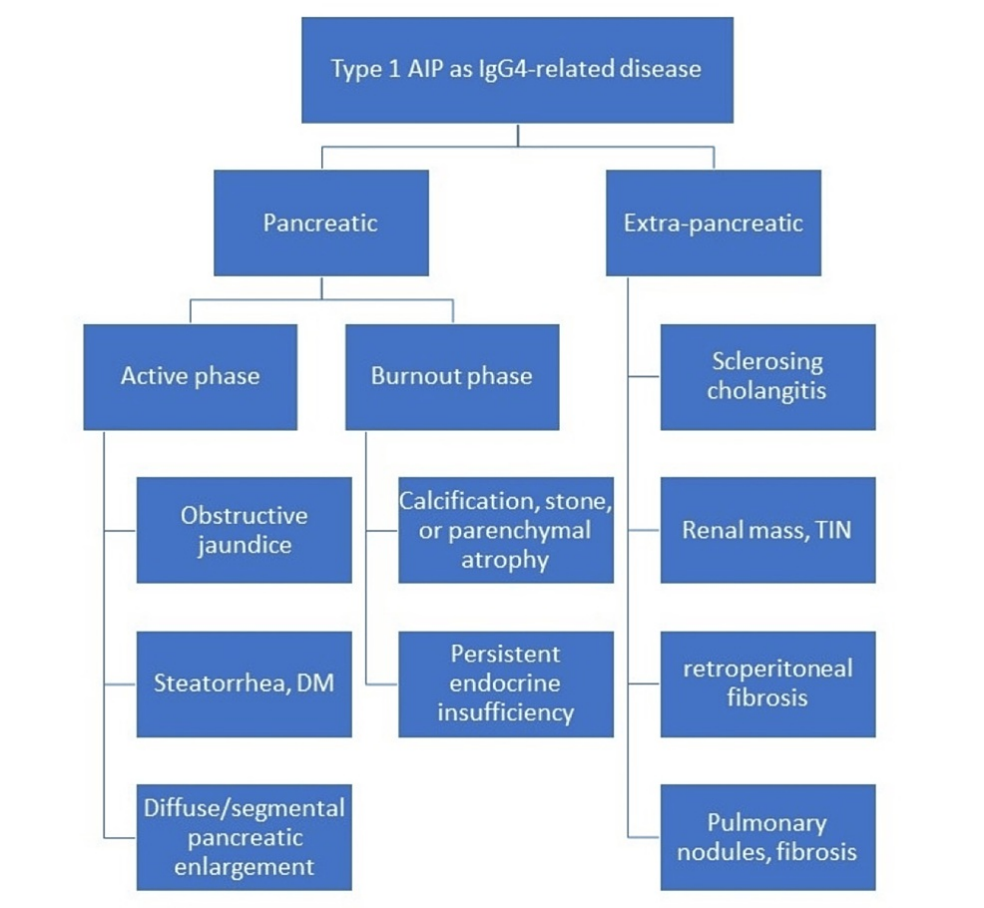
Pancreatic and extra-pancreatic manifestations of IGg4-related disease. AIP: autoimmune pancreatitis, TIN: tubular interstitial nephritis.

AIP is a rare form of chronic pancreatitis characterized by a substantial lymphoplasmacytic infiltrate, fibrosis, and obliterative phlebitis in the pancreatic tissue. AIP is categorized into two subtypes: Type 1 and Type 2 [5]. The first classification is the type I AIP, also referred to as lymphoplasmacytic sclerosing pancreatitis (LPSP). Type I AIP tends to manifest in later adulthood and is generally diagnosed at 50 years and above. This form is more prevalent in males, occurring three times more frequently than in females [4]. Type 1 AIP may serve as a facet within the spectrum of IgG4-related disease (IgG4-RD), characterized by multi-organ involvement and extrapancreatic manifestations with involvement of eyes, bile ducts, lymph nodes, salivary glands, thyroid, kidneys, and lungs (Figure 3) [6]. AIP has a favorable prognosis if diagnosed early, and treatment is initiated. Relapse occurs only in 31% of patients with Type 1 AIP as compared to type 2 AIP [7].

**Figure 4:**
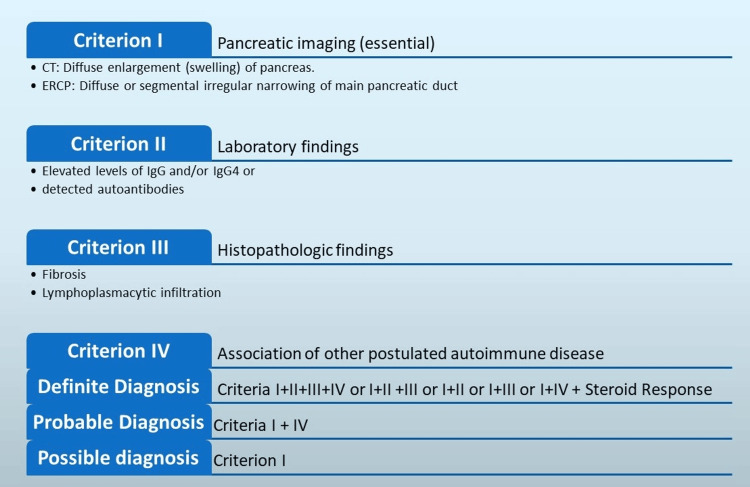
Diagnostic criteria for AIP diagnosis. CT: computed tomography, ERCP: endoscopic retrograde cholangiopancreatography, Ig: Immunoglobulin, AIP: autoimmune pancreatitis. Image credit: Adesola A. Agboola, Khalid H. Mohamed

Patients with AIP might exhibit no symptoms or experience mild abdominal discomfort without pancreatitis episodes (Table 1) [8]. The diagnosis of AIP particularly primarily relies on histopathology, as serological markers alone may not be sufficient [9]. EUS-FNB played a pivotal role in obtaining a definitive tissue diagnosis by demonstrating characteristic histopathological features of AIP. Measuring serum IgG4 levels can provide valuable support for the diagnosis of AIP [10,11]. In this case, the markedly elevated IgG4 levels were consistent with Type 1 AIP and further reinforced the diagnosis. Many criteria for AIP diagnosis were proposed for diagnosing AIP, which are shown in Figure 4 [12-15].

**Table 1:** Percentage of symptoms presenting in AIP. N: number.

Symptoms	N	Frequency (%)
Obstructive jaundice	33	33
Abdominal pain	32	32
Backache	15	15
Weight loss	15	15
Anorexia	9	9
Fatigue	9	9
Change in bowel habits	7	7
Fever	6	6
Asymptomatic	15	15

The management of AIP primarily involves immunosuppressive therapy with corticosteroids with 30-40 mg, or 0.6 mg/kg, per day of prednisolone for two to four weeks with careful monitoring of clinical features and serological and imaging findings [16]. Resolution of clinical features generally begins within two weeks, and clinical remission is generally observed within four weeks of treatment. Therefore, guidelines recommend tapering steroids every two weeks over two months, and a maintenance dose is maintained for six months to three years based on the response [17]. In non-responsive patients, a combination of immunomodulatory agents such as rituximab, azathioprine, or mycophenolate can be used [18]. Biliary stenting is recommended in obstructive jaundice, either benign or malignant cause [19]. In our patient, corticosteroid treatment led to a rapid improvement in clinical symptoms. It is crucial to initiate treatment promptly to alleviate symptoms, prevent complications, and improve the patient's overall quality of life.

## Conclusions

Our case highlights the diagnostic challenges of autoimmune pancreatitis, especially when it presents obstructive jaundice and radiological features that mimic pancreatic cancer. The clinical and radiological similarities between AIP and pancreatic cancer emphasize the importance of a thorough diagnostic evaluation, including tissue sampling. Our case highlights the significance of timely and accurate differentiation from pancreatic cancer and emphasizes the need for comprehensive evaluation, multidisciplinary collaboration, and management of this challenging clinical scenario.
